# Varieties of felt presence? Three surveys of presence phenomena and their relations to psychopathology

**DOI:** 10.1017/S0033291722000344

**Published:** 2023-06

**Authors:** Ben Alderson-Day, Peter Moseley, Kaja Mitrenga, Jamie Moffatt, Rebecca Lee, John Foxwell, Jacqueline Hayes, David Smailes, Charles Fernyhough

**Affiliations:** 1 Department of Psychology, Durham University, Durham, UK; 2 Department of Psychology, Northumbria University, Newcastle Upon Tyne, UK; 3 Department of Psychology, University of Sussex, Falmer, UK; 4 Hull York Medical School, University of Hull, Hull, UK; 5 Department of Psychology, University of Roehampton, London, UK

**Keywords:** Bodily self, dissociation, hallucinations, psychosis, social cognition

## Abstract

**Background:**

Experiences of felt presence (FP) are well documented in neurology, neuropsychology and bereavement research, but systematic research in relation to psychopathology is limited. FP is a feature of sensorimotor disruption in psychosis, hypnagogic experiences, solo pursuits and spiritual encounters, but research comparing these phenomena remains rare. A comparative approach to the phenomenology of FP has the potential to identify shared and unique processes underlying the experience across these contexts, with implications for clinical understanding and intervention.

**Methods:**

We present a mixed-methods analysis from three online surveys comparing FP across three diverse contexts: a population sample which included people with experience of psychosis and voice-hearing (study 1, *N* = 75), people with spiritual and spiritualist beliefs (study 2, *N* = 47) and practitioners of endurance/solo pursuits (study 3, *N* = 84). Participants were asked to provide descriptions of their FP experiences and completed questionnaires on FP frequency, hallucinatory experiences, dissociation, paranoia, social inner speech and sleep. Data and code for the study are available via OSF.

**Results:**

Hierarchical linear regression analysis indicated that FP frequency was predicted by a general tendency to experience hallucinations in all three studies, although paranoia and gender (female > male) were also significant predictors in sample 1. Qualitative analysis highlighted shared and diverging phenomenology of FP experiences across the three studies, including a role for immersive states in FP.

**Conclusions:**

These data combine to provide the first picture of the potential shared mechanisms underlying different accounts of FP, supporting a unitary model of the experience.

## Introduction

Characterised by a basic feeling that someone is present in the immediate environment without any clear sensory content (Critchley, 1955; Jaspers, 1913), felt presences (FP) occur in survival situations, bereavement and hypnagogia (Hayes & Leudar, 2016; Kamp et al., 2020; Nielsen, 2007; Suedfeld & Geiger, 2008); present in neurological disorders including epilepsy and Parkinson's disease (Brugger, Regard, & Landis, 1997; Reckner, Cipolotti, & Foley, 2020); and can be induced via neurostimulation and virtual reality (Arzy, Seeck, Ortigue, Spinelli, & Blanke, 2006; Erickson-Davis et al., 2021). Currently unclear is whether these unusual and varied experiences share common foundations or represent the same underlying phenomenon.

FP are described in psychosis case reports (Jaspers, 1913) and accounts of extracampine hallucinations (Bleuler, 1903), but psychiatric investigations are relatively limited (Critchley, 1955). Recently FPs have begun again to be recognised as one of many complex experiences occurring within psychosis. A variety of disruptions to the bodily self have been described in schizophrenia (Benson, Brugger, & Park, 2019), while phenomenological work on auditory verbal hallucinations (AVH) has described FP in relation to voice-hearing^†^^1^, with some voices experienced as ‘present’ even when silent (Woods, Jones, Alderson-Day, Callard, & Fernyhough, 2015). One study reported an incidence of 52% for FP in voice-hearers with early psychosis (Alderson-Day et al., 2021).

FPs are often treated as a kind of hallucination, but in psychosis the lack of sensory content could suggest that presences are instead a kind of delusion. FP could also be a secondary consequence of hallucinations or passivity; a post-hoc inference that ‘someone is here’ when faced with other signs of agency. If so, FP may associate with trait measures of paranoia, beyond any general association with hallucinations. Assuming a continuum model (Van Os, 2003), this could be evident in clinical and non-clinical populations.

Alternatively, given the range of non-self and embodied experiences associated with dissociation (Carlson & Putnam, 1993), FP may be better characterised as a dissociative state akin to depersonalisation. That is, a presence could plausibly arise from changes to how the parts and boundaries of one's own body are recognised, as can occur for various autoscopic phenomena (Brugger et al., 1997). There is evidence to suggest that FP may be differentiated from some major disruptions to bodily self-awareness, such as out-of-body (OBE) experiences (Blanke et al., 2005; Cheyne & Girard, 2007). However, many FP accounts involve a feeling of connection to the perceived presence (Geiger, 2009), and its positioning may even mirror that of the perceiver (Arzy et al., 2006). The potential link to feelings of depersonalisation about one's own body would therefore seem to pose a *prima facie* case for further investigation. Understanding presence as a form of depersonalisation would fit with its occurrence in survival accounts, in which presence and other autoscopic phenomena regularly occur under stress and at the limits of endurance (Suedfeld & Geiger, 2008).

Potentially relevant non-psychopathological factors include social imagery and sleep disruption (Nielsen, 2007). Regarding the former, some studies of bereavement conceptualise FP as a kind of continued relationship (Hayes & Leudar, 2016). Imagining speech involving others' voices is one example of representing a kind of illusory ‘other’ (McCarthy-Jones & Fernyhough, 2011), whose identity typically reflects life experiences and important relationships. A vivid inner social world could therefore relate to FP in healthy and clinical samples alike (Nielsen, 2007). Sleep disruption represents another potential factor given its associations with psychotic and hypnagogic experiences including sleep paralysis (Nielsen, 2007), and situations of extreme stress, such as polar expeditions (Suedfeld & Geiger, 2008).

In three online surveys, we set out to explore a range of presence experiences – their phenomenology, who has them and their correlates – deploying measures relating to psychopathology, social imagery and sleep. First, we invited members of the general public to share FP accounts (study 1). To explore FP's relations to AVH, this included a specific invitation to individuals identifying as voice-hearers. We followed this with contrasting samples from two populations who also report FP: people who experience spiritual presences (study 2), and practitioners of extreme sports and solo pursuits (study 3). While previous research has examined specific elements of presence in similar samples (Barnby & Bell, 2017), to our knowledge this is the first phenomenological survey to take a comparative view across multiple contexts.

## Method

### Participants

In study 1, adults over 18 were invited to participate via social media and a project website (www.hearingthevoice.org). Seventy-five participants [Age_M(s.d.)_ = 39.10 (12.47) years] responded (see online Supplementary Table S1 for full demographics). Fifty-eight spoke English as their first language, with most participants (50/75) coming from the UK or USA. Reflecting the main recruitment route, a large minority (34/75) reported having received a psychiatric or neurological diagnosis (the most common being schizophrenia, 11/34), and 25 self-identified as a ‘voice-hearer’.

For study 2, we recruited via spiritual organisations including the Spiritualists National Union [*N* = 47, Age_M(s.d.)_ = 57.02 (10.61) years]. Most (30/47) were based in the UK and spoke English as their first language (38/47). Almost half identified as voice-hearers but only six reported clinical diagnoses, the most common of which was ADHD (*n* = 2). In total, 46/47 described having had clairaudient experiences in the past (i.e. involving verbal messages), 43/47 clairvoyance (visions) and 44/47 clairsentience (feelings or sensations).

For study 3 [*N* = 84, Age_M(s.d.)_ = 43.32 (11.49) years], recruitment took place via email lists and societies that promoted solo, endurance and extreme sports activities. Most (66/84) spoke English as their first language and were based in the UK or USA (55/84). Similar to study 2, diagnosis rates were generally lower than sample 1, and featured PTSD (*n* = 3) and depression (*n* = 4), including five participants identifying as voice-hearers. The most common activities reported were diving (48), climbing (27), mountaineering (25), caving (24), cycling (21), running (19), walking (17), sailing (15) and swimming (15).

### Measures

**Fig. 1.**
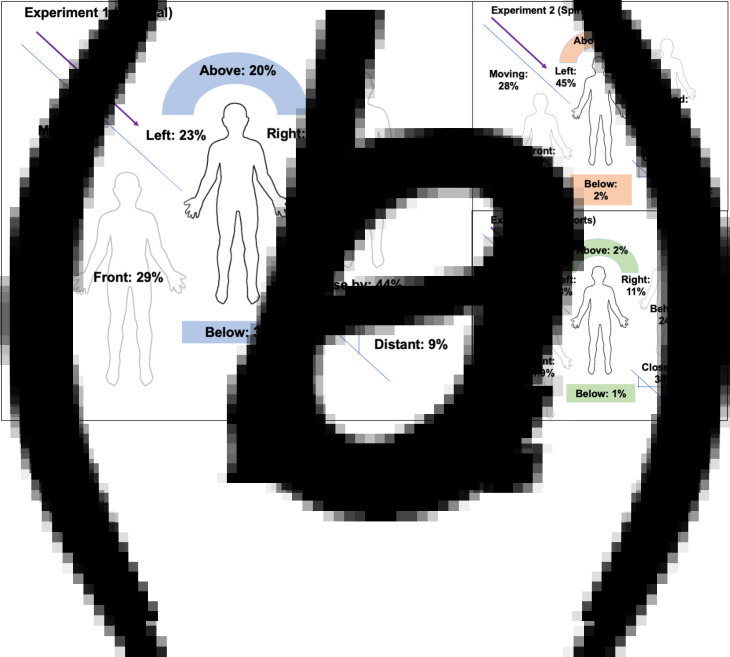
Spatial and contextual characteristics of presences in experiment 1 (*a*), experiment 2 (*b*) and experiment 3 (*c*).

Each sample received the same questionnaires via the online survey. To gather data on the frequency and nature of FP, we used:

- The presence subscale of the Multimodality Unusual Sensory Experiences Questionnaire – MUSEQ (Mitchell et al., 2017), which includes four items assessing FP frequency rated on a five-point Likert scale (Never–Frequently);
- Five questions about the kinds of presence experienced, selected from the PatientsLikeMe extracampine hallucinations questionnaire (Wood, Hopkins, Moodley, & Chan, 2015), a survey on presence hallucinations used in people with Parkinsonian disorders;
- Two free-text, open-ended questions about FP: (1) ‘Please describe your experiences of a feeling of a presence, in your own words’, and (2) ‘Please describe the context or situation you were in when you had a feeling of a presence’;
- Additional questions that collected quantitative data on presence location (see Fig. 1).

To assess predictors of FP experiences, we included:

1. The 11-item version of the Revised Launay-Slade Hallucination Scale (LSHS; McCarthy-Jones & Fernyhough, 2011), focusing on auditory experiences (five items) and visual experiences (four items);
2. The Paranoia Checklist (PC; Freeman et al., 2005), an 18-item scale assessing proneness to paranoid and delusional ideation;
3. The depersonalisation/derealisation subscale of the Dissociative Experiences Scale – Version 2 (DES; Carlson & Putnam, 1993), consisting of six items relating to unusual non-self experiences;
4. The ‘Dialogic’ and ‘Other People’ subscales of the Varieties of Inner Speech Questionnaire (VISQ; McCarthy-Jones & Fernyhough, 2011) selected as a proxy of social imagery;
5. The Sleep Condition Indicator (SCI; Espie et al., 2014), a measure of sleep quality and sleep problems.

### Data analysis

Quantitative variables were analysed using Spearman's correlation (due to non-normal distributions on almost all variables), non-parametric partial correlation tests using the ‘ppcor’ R package, and hierarchical regression. Residuals >2 s.d. and Cook's distance were used to identify outliers and influential cases. *B* values represent standardised beta values.

Directed content analysis (Hsieh & Shannon, 2005) was used to analyse free-text descriptions in each study. For study 1, each rater read the full dataset, generated an initial list of codes and discussed their codes with the coding team. A coding scheme was then applied to 20% of the data, tested for reliability and then applied to the remaining dataset. Coding for studies 2 and 3 began with the coding frame from study 1, adding new codes based on the content of each sample. Reliability was checked using Krippendorff's *α* for studies 1 and 3; for study 2, the sample was smaller and therefore fully co-coded by each rater and disagreements resolved. Log odds ratios centred on 0 were generated to examine differences in coding frequency by sample and other factors. They are included here for descriptive purposes due to the limited sample sizes. For clarity, in the text, we only focus on odds ratios with confidence intervals not crossing zero (Alderson-Day et al., 2021).

## Results

### Characteristics of felt presence (study 1)

Table 1 displays the FP self-report ratings on the MUSEQ and PatientsLikeMe scales, indicating that 38.7% of participants experienced frequent FPs. Frequent presences were described as angelic or malevolent at similar rates (9.3% *v.* 10.7%), more likely to be human than not, and familiar to individuals 58.7% of the time. Surprisingly, presences were also described as being sensed via touch in half the sample and being heard by nearly as many. Presences were most likely to be placed behind the person (50%) and close by (44%) (Fig. 1*a*).

**Table 1.** Felt presence frequency and characteristic ratings for study 1

	Frequency (%)				
	Never	Hardly ever	Rarely	Occasionally	Frequently
(a) *MUSEQ FP subscale*					
(i) I felt the presence of someone, even though I could not see them (e.g. behind me, or in another room).	9.3	18.7	12.0	21.3	38.7
(ii) I have felt an unseen evil presence around me.	36.0	34.7	6.7	12.0	10.7
(iii) I have felt an unseen angelic presence around me.	42.7	26.7	9.3	12.0	9.3
(iv) I have felt the presence of a relative or friend who has passed away.	38.7	26.7	18.7	9.3	6.7
(b) *Qualities of presence (PatientsLikeMe)*					
(v) Do you feel as though this presence is human?	Human	58.7	Non-human	34.7	
(vi) Is the presence familiar to you?	Yes	58.7	No	32.0	
(vii) Does the presence ever speak to you or make another noise?	Yes	46.7	No	46.7	
(viii) Does the presence ever touch you?	Yes	50.7	No	42.7	
(ix) Are these experiences always the same?	Yes	25.3	No	66.7	

Sixty-three participants completed free-text descriptions of the presences they experienced (Box 1). Descriptions could be relatively minimal (‘It feels like someone is standing behind me and watching me.’) and sometimes experienced with a specific valence (‘A feeling of utter terror, as if this presence had the power to hurt me.’). Codes for those descriptions are displayed in Table 2, indicating that FPs often had a distinct *identity*, were experienced in *indoor* settings, and were described as simply been *felt or known* to be there without any other sensory cues.

**Table 2.** Felt presence characteristics from free-text responses in studies 1, 2 and 3

Code	Definition	Study 1 (population) (%)	Study 2 (spiritual) (%)	Study 3 (sports) (%)	logOR
Auditory/verbal	Specific content relating to sound and language	34.9	25.6	12.8	1 > 3: 1.28
Visual	Presences described with visual outlines/cues	22.2	27.9	25.5	
Tactile/bodily	Body felt to be changed or touched	25.4	37.2	14.9	2 > 3: 1.22
Smell	Specific content relating to smell	4.8	2.3	4.3	
Personal space	Explicitly described in peripersonal space	27.0	44.2	21.3	2 > 3: 1.07
Fear/dread	Presences described as scary, frightening	25.4	9.3	19.1	1 > 2: 1.18
Warmth/comfort	Experience described as comforting, pleasant	28.6	37.2	27.7	
Familiarity	Familiar/ previously known to person	34.9	32.6	29.8	
Identity/form	Distinct identity (human, angel)	57.1	62.8	46.8	
Being watched/followed	Feeling FP is watching you	14.3	2.3	19.1	3 > 2: 2.30
Sense of purpose	Feeling FP is present for a reason/function	20.6	32.6	31.9	
Knowing/feeling	FP ‘just’ felt or known to be present	44.4	62.8	36.2	2 > 3: 1.09
Grief/bereavement/death	Associated with past bereavement	28.6	18.6	25.5	
Stress/illness	Linked to period of stress/illness	27.0	7.0	12.8	1 > 2: 1.57
Multiple – single context	Occurs multiple times in same/similar context	41.3	25.6	48.9	3 > 2: 1.03
Multiple – varied contexts	Occurs multiple times in dissimilar contexts	34.9	65.1	17.0	2 > 1: 1.27; 1 > 3: 0.94; 2 > 3: 2.21
Immersion	Occurs when fully absorbed in an activity	11.1	34.	2.1	2 > 1: 1.47; 2 > 3: 3.20
Interaction	Can be interacted with (spoken to, touched)	14.3	27.9	6.4	2 > 3: 1.74
Spiritual	Experience or identity spiritual in nature	20.6	60.5	4.3	2 > 1: 1.79; 1 > 3: 1.75; 2 > 3: 3.54
Sleep	Occurs around boundaries of sleep	36.5	18.6	27.7	
Mundane	Experience described as overly familiar, everyday	25.4	–	12.8	
Inside^a^	Specifically linked to particular indoor space	46.0	69.8	21.3	2 > 1: 1.02; 1 > 3: 1.12; 2 > 3: 2.14
Outside^a^	Specifically linked to particular outdoor space	9.5	14.0	66.0	3 > 1: 2.93; 3 > 2: 2.48
Study 2: involuntary	Specifically noted as unexpected	–	72.10	–	
Study 2: spiritualist practice	Experienced part of work as medium	–	41.90	–	
Study 3: physical tiredness	Occurrence explicitly linked to exhaustion	–	–	27.70	
Study 3: sports activity	FP linked to specific sports activity	–	–	57.40	
Study 3: risk of death	FP occurs only when in serious danger	–	–	12.80	

^a^Indoor and outdoor codes were not mutually exclusive.

Box 1. Examples of presence

‘I experience a sense of presence about once every few weeks. It is as real as turning to acknowledge another person only to find there is no-one there. The sense of presence can be weaker or stronger and tends to fade and disappear after a brief period, usually from a few moments to a few minutes. I first noticed the experience when I was in my early twenties (I'm now in my late thirties). In the past 5 years or so I've experienced it more frequently. Over the past year it's been accompanied by a slight sense of panic and disorientation.’

[Study 1, P53. Example codes: *Knowing/Feeling, Familiarity*]

‘It is like the personification of a feeling I have and is related to the particular context I am in, these come from within me. Others feel more like what people describe as a haunting, they come from outside and are about things that are not about me just now. I just feel that it is there and what qualities the presence has. Sometimes it feels like I should be able to see it or hear it as I am looking at it, but there is nothing there or it seems to be moving but there is no sound. I can feel certain that it is there while seeing that it is not there and being equally certain that there is nothing there to see. The feeling may arrive suddenly or I may feel the presence creeping up more slowly’.

[Study 1, P54. Example codes: *Identity/Form, Multiple Different Contexts*]

‘The very first time; in bed, feeling that I am awake but unable to move and completely aware that there is a malevolent presence moving towards me and I can't move or look at it or even scream. I managed to get my fingers to move then I could move and it went. But it completely freaked me out and left me terrified that night…they came nightly for months. I learnt to wake myself up by repeatedly trying to speak and as soon as I actually vocalised something I could move/wake. I had these on a very regular occurrence for over a year. In the last 10 years it has probably happened fewer than 5 times.’

[Study 1, P10. Example codes: *Sleep, Fear/Dread, Multiple Occurrences in Same Context*]

‘I awoke from sleep to feel the presence of my late ex-boyfriend standing in the middle of the room. He was not visible to me, but I knew he was there, and that it was him rather than anyone else. About 18 months later, I felt his presence in bed beside me. Again I could not see him, but I could feel the pressure of his body along the length of mine.’

[Study 1, P2. Example Codes: *Tactile, Personal Space, Familiarity*]

‘The last time I was sat at my desk concentrating on my work and I felt spirit touch me at the back of my head. It's a comforting experience, not at all disturbing.’

[Study 2, P2. Example Codes: *Warmth/Comfort, Inside, Spiritual*]

‘I know the person whose presence I experience. She is a young woman with her own problems and life story which is completely different from and separate from me and my life, yet we are linked. Sometimes there are words, images and always emotions that I pick up from her. Sometimes I can see her as an overlay on my normal vision. when this occurs there is a background around her of her surroundings which are different from those in my flat.’

[Study 2, P4. Example Codes: *Visual, Identity/Form, Purpose*]

‘I had succumbed to hypothermia in a race and had basically decided to sleep in some marshalls and give up. Something told me to open my eyes and ahead I saw a light. I sprung back into life and found my way back to the finish of the race. If I hadn't I would be dead no question about it.’

[Study 3, P7. Example Codes: *Purpose, Physical Tiredness*]

‘The feeling that someone is watching me, not necessarily for my benefit either. [It occurs] in an old mine, usually when I'm alone or at the rear of a party.’

[Study 3, P17. Example Codes: *Being Watched, Outside*]

N.b. Examples do not represent complete responses in all cases (due to length).

### Associations with psychopathology (study 1)

To explore associations with FP, we summed the MUSEQ items to give a total ‘FP score’ (Cronbach's *α* = 0.69). Spearman's correlations indicated that, apart from Dialogic VISQ (*r* = 0.23, *p* = 0.05), all of the other trait predictors (LSHS, PC, DES, VISQ-O and SCI) were significantly associated with FP score (all *r* > 0.35, all *p* < 0.002; see online Supplementary materials). However, there was also considerable intercorrelation amongst these variables, particularly for LSHS (*r* paranoia = 0.62, *r* DES = 0.83). Partial correlation analysis – controlling for LSHS – resulted in no other variables being significantly associated with FP, apart from DES scores (*r* = 0.31, *p* = 0.008). This suggested that a general propensity towards hallucinatory experiences fully mediated most associations with FP (all *r* < 0.38, all *p* > 0.05).

We then used regression modelling to establish a demographics model, a ‘psychopathology’ model (including LSHS, Paranoia and DES scores; model 1) and a ‘non-clinical’ model (adding VISQ and sleep scores; model 2). The demographics model [*F*_(6,65)_ = 3.82, *p* = 0.003, adj. *R*^2^ = 0.19] included significant associations for FP with Gender (*B* = −0.27, *p* = 0.0017, CI −0.49 to −0.05) and Diagnostic status (*B* = 0.35, *p* = 0.002, CI 0.13–0.57), such that men and those without a psychiatric diagnosis reported fewer presences^2^. Age (*B* = −0.14, *p* = 0.204) and Education (*p* values 0.50–0.86) made no significant contribution to the model. Gender and Diagnosis were therefore retained for a parsimonious demographics model to aid model comparison (model 0).

Initial results for model 1 indicated significant fit with a high proportion of variance explained [*F*_(5,66)_ = 13.73, *p* < 0.001, adj. *R*^2^ = 0.47], but only Gender and Paranoia making significant contributions, despite the high bivariate correlations observed previously (online Supplementary Table S3). Diagnostic tests indicated significant multicollinearity specific to hallucinations, paranoia, and dissociation, suggesting that at least one predictor needed to be removed. Given the primary relevance of hallucinations to our research question, and the very high LSHS-DES correlation, we removed DES and reran the model (model 1b). The resulting model explained significantly more variance than the demographics alone [Δ*R*^2^ = 0.32, *F*_(2,67)_ = 19.71, *p* < 0.001], with predictive associations now evident for Gender (*B* = −0.29, *p* = 0.001, CI −0.46 to −0.12), LSHS (*B* = 0.32, *p* = 0.010, CI 0.08–0.57), and paranoia (*B* = 0.32, *p* = 0.015, CI 0.06–0.58; online Supplementary Table S4).

Model 2 included Dialogic VISQ, Other People VISQ and sleep condition scores, but did not account for significantly more variance and identified no new significant predictors [*F*_(3,64)_ = 1.35, *p* = 0.266, Δ*R*^2^ = 0.0087]. It appeared to affect the overall power of the model – with paranoia and LSHS scores marginally significant and non-significant, respectively – but at the expense of parsimony (as indicated by rising AIC scores). Reverting to model 1, it was therefore apparent that general hallucination-proneness, paranoia and gender made independent contributions to FP occurrence.

### Voice-hearing and FP (study 1)

To explore FP's relations to AVH, odds ratios were generated for each code from the qualitative analysis. Voice-hearing was associated with *tactile* presences (lgOR = 1.29, CI 0.09–2.50). Non-voice-hearers in contrast were more likely to describe FPs as just *felt*/*known* to being there, i.e. in the absence of other senses (lgOR = −1.58, CI −2.76 to −0.41), and FPs occurring *multiple times* in the same context (lgOR = 1.38, CI 0.21–2.55). We ran a similar analysis to check for the influence of diagnosis, but no notable differences were observed (with all CIs crossing zero).

### Characteristics of FP in studies 2 and 3

Figure 1 and Table 2 display characteristics of FP reported in studies 2 and 3. In study 2, 43/47 participants provided free-text descriptions. Two additional codes were necessary to include (i) involuntary presences and (ii) spiritualist practices (FP came during deliberate practice in many cases, thus participants were keen to distinguish different contexts). In contrast, presences being experienced as *mundane* did not feature in sample 2. Although the *spiritual* code may be expected to apply for all of this sample, in practice participants reported a range of experiences, not all of which were described as being explicitly spiritual. For study 3, 47/84 wrote free-text descriptions. Codes added included references to a specific sporting activity, physical tiredness and dangerous situations.

While some codes were common in each sample – such as presences experienced as having a particular *identity* – odds ratios also indicated qualitative differences between the groups (Table 2). Predictably, *spiritual* presences were most associated with study 2, but they were also more common in sample 1 compared to the sports sample. A similar pattern was evident for presences occurring in *multiple different contexts*. In contrast, the feeling of *being watched* was more common in the sports sample than the spiritual sample.

Sample 1 was distinguished from the spiritual sample by being more associated with *stress and illness*, and feelings of *fear and dread*, while they differed from the sports sample by including more *auditory-verbal* experiences. FP in study 2 were most likely to be associated with states of *immersion* and were further distinguished from study 3 by being more likely to be *interactive*, *tactile*, positioned in *personal space* and recognised by a basic *feeling or knowing* that ‘someone’ was present.

### Associations with psychopathology (studies 2 and 3)

Although not our focus, Table 3 displays the mean scores for each predictor variable in samples 1, 2 and 3, showing that the groups varied considerably on all outcomes. Notably, DES scores for the spiritual and sports samples had few cases with a score >1 with the large majority of cases clustering at zero, rendering the measure redundant for assessing correlations in these two samples (see online Supplementary materials).

**Table 3.** Questionnaire scores for studies 1–3

	Study 1 (population)		Study 2 (spiritual)		Study 3 (sports)		Group difference	Pairwise differences
	M	s.d.	M	s.d.	M	s.d.		
Felt presence score	10.25	3.88	13.62	2.99	7.61	3.50	***	2 > 1 > 3
Hallucinations (LSHS)	15.15	5.39	13.09	3.36	11.68	3.41	***	1 > 3, 2 > 3
Paranoia (PC)	31.40	15.96	21.08	5.45	20.60	8.52	***	1 > 2, 1 > 3
Derealisation (DES)	1.80	2.12	0.61	1.07	0.14	0.88	***	1 > 2 > 3
Dialogic inner speech (VISQ-R)	15.55	7.20	12.75	6.31	12.04	6.40	**	1 > 3
Other people in inner speech (VISQ-R)	12.31	8.61	9.60	6.47	7.61	4.50	***	1 > 3
Sleep quality (SCI)	16.94	8.15	21.32	5.43	22.67	6.98	***	1 > 2, 1 > 3

***p < 0.001, **p < 0.01. All reported pairwise comparisons significant at at least *p* < 0.05, Bonferroni corrected.

Removing DES scores, the same analytic procedure as in study 1 was applied in studies 2 and 3. For study 2, pairwise correlations indicated significant associations with FP for the LSHS only (*r* = 0.44, *p* = 0.002). Regression analysis indicated an effect of diagnosis in the demographics model (*B* = 0.32, *p* = 0.036, CI 0.02 to –0.63). There was a significant effect of LSHS in model 1 (*B* = 0.31, *p* = 0.029, CI −0.05 to 0.68), but no other significant predictors. For study 3, FP score correlated with LSHS (*r* = 0.44, *p* < 0.001), Other People VISQ (*r* = 0.27, *p* = 0.014) and sleep condition scores (*r* = −0.29, *p* = 0.008), but regression modelling only identified LSHS scores as a consistent predictor of presence experiences (model 1: *B* = 0.54, *p* < 0.001, CI 0.33–0.75). Education was a significant predictor in the initial demographics model (*B* = 0.33, *p* = 0.043, CI 0.01–0.69).

## Discussion

Our main findings were that (i) FP frequency was consistently related to general hallucination-proneness, (ii) paranoia and gender significantly predicted FP in sample 1, and (iii) qualitative characteristics of presence differ across contexts and samples, but with large degrees of overlap.

The high rates of psychiatric diagnosis (>50%) and voice-hearing (25%) in our initial sample made study 1 the most relevant for considering potentially pathological FP. This sample was unique in showing significant roles for both hallucination-proneness and paranoia, suggesting that feelings of persecution are important for understanding FP. Self-identifying voice-hearers reported more presences involving tactile cues and bodily changes, consistent with the reports of voice-hearing emphasising voices' embodied nature, including how tactile cues can be associated with anticipating voice occurrence (Woods et al., 2015). It also coheres more broadly with an emerging literature on body disruptions in psychosis (Benson et al., 2019) and fluidity in agency judgements when presences are induced (Orepic, Rognini, Kannape, Faivre, & Blanke, 2021). Notable also was a distinct gender effect in this sample, with female participants much more likely to report FP in study 1. Given that AVH are often perceived as male, with aggressive and persecutory characteristics (Nayani & David, 1996), an important avenue for clinical research may be to explore whether women with psychosis are particularly affected by intrusive FP, including the possibility of this having social/relational origins.

In studies 2 and 3, we anticipated that other factors such as social imagery or poor sleep would predict FP. Instead, we found that only general hallucination-proneness predicted FP, suggesting that, among various populations and groups, FP may simply reflect another kind of hallucinatory state. More surprisingly, we also observed very low dissociation scores in these samples, despite the fact that dissociative processes offer a plausible framework to link unusual presence-like phenomena in a variety of contexts (Luhrmann et al., 2019; Suedfeld & Geiger, 2008).

Our qualitative results indicate why this might be. In sample 2, participants from spiritual communities were more likely to describe presences that occurred during a state of ‘immersion’, i.e. when fully engrossed in a focused mode of internal attention. This is comparable to absorption (Tellegen & Atkinson, 1974), which has been proposed recently as a key process by which spiritual and hallucinatory experiences can be cultivated (Erickson-Davis et al., 2021; Luhrmann et al., 2021), and is sometimes considered a form of dissociation. We measured depersonalisation in the DES because of its direct relevance to unusual sensory experiences, but it is possible that absorption measures could instead have picked out FP occurrence.

Our qualitative coding also identified key points of difference and similarity between the varieties of presence described. FP in sample 1 were more likely to be frightening and associated with illness, but FP experiences reported by sample 3 were most likely to feature a feeling of being watched. FP in the context of our sports sample were less likely to be spiritual and tended to occur in the same context when outside – as would be expected for generally non-recurrent phenomena in a predominantly non-clinical group. Nevertheless, our analyses suggested that there were more similarities than differences in the types of FP experiences reported by the three samples. Participants in each group described presences with a specific form and identity, and examples relating to bereavement were evident throughout (either as a trigger or as reflected in FP content). The link between FP content and bereavement implies an existing relationship with the ‘presence’ and therefore some form of continuity with a previous relational context. In addition, in all three samples, FP occurred when people were falling asleep or waking up.

There are important limitations to consider when interpreting these data. First, they are cross-sectional, so establishing causality is impossible. Second, the collinearity evident in the data in study 1 shows how closely hallucinations, paranoia and dissociation interrelate. Further work is required in larger clinical groups to establish how these factors can be separated. Experience-sampling may be useful for investigating how feelings and experiences of threat and depersonalisation dynamically contribute to hallucinatory perceptions of another entity. Finally, all data were gathered online, and we therefore cannot verify reported diagnoses, nor explore FP descriptions in depth. Diagnostic status did not appear to greatly affect FP phenomenology (compared to voice-hearing, e.g.), but face-to-face interview methods will be an important next step in exploring the phenomenology and clinical relevance of FP. This is particularly crucial given the difficulties individuals have in describing FP, which can often vary considerably in their distinctiveness and abstract nature (Hayes & Leudar, 2016). It is possible that in-depth interviewing would also allow for more fine-grained distinctions to be made between different changes to the experience of self and body, enabling differentiation between FP and other unusual experiences such as feelings of depersonalisation. A custom interview, or a phenomenological tool such as the Examination of Anomalous Self-Experience (EASE) scale (Parnas et al., 2005) may be required for future research on the topic.

Another important consideration here concerns the framing and interpretation of FP in relation to psychopathology, when alternative approaches may be crucial to hold and explore. The associations of FP with gender and tactile experiences for voice-hearers may suggest a role for trauma in the origin and recurrence of unwanted presences. Future studies could pursue this question with the inclusion of appropriate measures of trauma and adversity. Our main finding here – that FP can be considered a kind of hallucination – would seem to categorise the experience as pathology, but it is important to recognise that many such experiences are proposed to arise from non-pathological origins. Indeed, given the range of FP experiences reported, it is clear that their impact may not be pathological at all. Recurring FP may reflect life experiences or – in some cases – a fundamental orientation to being with others. Understanding the nature of FP likely requires dialogue across several different interpretative frameworks: for instance, feminist approaches to topics such as grief offer an alternative and non-pathologising lens by which to understand certain kinds of persisting FP (Granek, 2021).

Notwithstanding the above, it is nevertheless possible to posit a range of predictions with relevance to FP more broadly. First, many presences are characterised by perceptions of a specific ‘other’, with spatial location, and with bodily/tactile characteristics. It seems likely that people prone to FP are more susceptible to experimental inductions of presence (Blanke et al., 2014), and changes to agency judgements following such interventions (Orepic et al., 2021). In other words, many instances of FP will result from disruption to bodily self-awareness. Second, context will shape the character of FP, with identity and valence varying across clinical and non-clinical groups. For example, presences during endurance sports may result from disruptions to bodily self-awareness, but actually be experienced as ‘being watched’ due to the context of being alone and outside. It remains to be seen whether absorption contributes to either of these processes, or instead represents a second route towards FP which is more characterised by intense states of imagination, as seen in readers' and writers' (Foxwell, Alderson-Day, Fernyhough, & Woods, 2020) experience of fictional characters with a vivid sense of presence. Given that some FP lack clear embodied characteristics, the possible existence of other kinds of illusory presence demands further investigation.

## Footnotes

† The notes appear after the main text.

## Notes

1 In contrast to auditory hallucinations, voice-hearing is often a preferred term used by people with lived experience.

2 Due to the small number of participants reporting their gender as ‘Other’, these cases were removed from regression analysis, resulting in an *n* of 72 for sample 1.

## Supplementary material

For supplementary material accompanying this paper visit https://doi.org/10.1017/S0033291722000344. click here to view supplementary material
