# Supplementary material for: Varieties of felt presence? Three surveys of presence phenomena and their relations to psychopathology
Source: Psychol Med. 2022 Mar 1;53(8):3692–700. doi: 10.1017/S0033291722000344 (PMC10277754; doi:10.1017/S0033291722000344)
Supplement: Supplementary file 1 [file S0033291722000344sup001.pdf]

Supplementary Materials for **Varieties of felt presence? Three surveys of presence phenomena and their relations to psychopathology.**

Table 1. Demographics for each sample

		Sample 1 (General)	Sample 2 (Spiritual)	Sample 3 (Sports)
**Gender**	**Female**	51	28	26
	**Male**	18	13	56
	**Other**	3	2	1
	Not stated	3	4	1
**Based in**	**UK**	36	30	45
	**USA**	14	4	10
	Other (all N<5)	25	13	29
**Education**	**Postgraduate**	36	15	26
	**Undergraduate**	27	10	38
	**A-Level**	4	5	7
	**GCSE**	5	14	13
	Not stated	3	3	0
**Voice-hearer?**	**Yes**	25	21	5
	**No**	49	26	78
	Not stated	1	0	1
**Diagnosis**	**Yes***	34	6	12
	Schizophrenia	11	1	0
	Depression	8	0	4
	BD	6	0	0
	PTSD	5	1	3
	DID	3	0	0
	Anxiety	3	0	1
	Epilepsy	2	0	0
	ADHD	1	2	0
	ASD	0	0	1
	Eating disorder	1	0	0
	PD	1	0	0
	Migraine	1	0	0
	M.E.	0	1	0
	M.S.	0	0	1
	TBI	0	0	1
	Other (unspecified)	0	1	2
	**No**	37	40	70
	**Not stated**	4	1	2

*Note multiple diagnoses could be selected by participants.

Table 2: Correlation matrix for sample 1

	LSHS	Paranoia Checklist	DES Derealisation	VISQ Dialogic	VISQ Other	SCI
Felt Presence (MUSEQ)	0.55***	0.47***	0.60***	0.23	0.42***	-0.36**
LSHS	1	0.62***	0.81***	0.56***	0.54***	-0.53***
Paranoia Checklist	-	1	0.63***	0.31**	0.48***	-0.29*
DES Derealisation	-	-	1	0.58***	0.58***	-0.33***
VISQ Dialogic	-	-	-	1	0.41***	-0.13
VISQ Other	-	-	-	-	1	-0.17

Partial correlations between felt presence and other variables, controlling for LSHS: 1) paranoia ($r = 0.20$, $p = 0.096$), 2) DES ($r = 0.31$, $p = 0.008$), VISQ Dialogic ($r = -0.12$, $p = 0.32$), VISQ Other ($r = 0.16$, $p = 0.162$), sleep condition indicator ($r = -0.09$, $p = 0.45$).

Table 3. Regression statistics for study 1

		Beta	SE	*B*	t	p	Low CI	High CI	F	df	adj. R2	p
**Model 0**	Gender (Male)	-2.85	0.96	-0.32	-2.97	0.004	-0.53	-0.10	9.26	2,69	0.15	<0.001
	Diagnosis	2.77	0.84	0.35	3.30	0.002	0.14	0.57			**AIC:**	390.71
**Model 1**	Gender (Male)	-2.51	0.79	-0.28	-3.18	0.002	-0.45	-0.10	13.73	5,66	0.47	<0.001
	Diagnosis	0.63	0.77	0.08	0.82	0.416	-0.12	0.28			AIC:	362.39
	LSHS[a]	0.17	0.11	0.24	1.59	0.116	-0.06	0.55				
	Paranoia[a]	0.07	0.03	0.30	2.31	0.024	0.04	0.56				
	DES[a]	0.23	0.26	0.13	0.91	0.367	-0.16	0.42				
**Model 1b**	Gender (Male)	-2.62	0.78	-0.29	-3.38	0.001	-0.46	-0.12	17.00	4,67	0.47	<0.001
	Diagnosis	0.78	0.75	0.10	1.03	0.305	-0.09	0.29			**AIC:**	361.40
	LSHS	0.23	0.09	0.32	2.50	0.010	0.08	0.57				
	Paranoia	0.08	0.03	0.32	2.64	0.015	0.06	0.58				
**Model 2**	Gender (Male)	-2.80	0.78	-0.31	-3.59	0.001	-0.49	-0.14	10.44	7,64	0.48	<0.001
	Diagnosis	0.41	0.81	0.05	0.50	0.616	-0.16	0.26			**AIC:**	362.98
	LSHS	0.15	0.11	0.21	1.46	0.151	-0.08	0.51				
	Paranoia	0.07	0.03	0.29	2.24	0.029	0.03	0.55				
	VISQ-Dialogic	-0.04	0.06	-0.07	-0.63	0.530	-0.27	0.14				
	VISQ-Other	0.08	0.05	0.17	1.53	0.130	-0.05	0.40				
	SCI	-0.07	0.05	-0.15	-1.41	0.164	-0.37	0.06				

a. Variables flagged as multicollinear on Klein test.
LSHS = Launay-Slade Hallucination Scale. SCI = Sleep Condition Indicator. VISQ = Varieties of Inner Speech Questionnaire.
AIC = Akaike Information Criterion (lower values = greater parsimony). Gender factor level is contrasted with “Female”.

Table 4 – Odds ratios by voice-hearing status in study 1.

	logOR	LoCI	HiCI
Interaction	1.47	-0.03	2.97
Smell	1.31	-1.15	3.77
Tactile	1.29	0.09	2.50
Being Watched	0.92	-0.52	2.35
Purpose	0.91	-0.33	2.15
Spiritual	0.91	-0.33	2.15
Sleep	0.88	-0.19	1.96
Grief/Bereavement	0.79	-0.32	1.91
Auditory/Verbal	0.71	-0.37	1.78
Multiple- Various	0.71	-0.37	1.78
Personal Space	0.41	-0.75	1.57
Stress-Illness	0.27	-0.87	1.41
Inside	0.22	-0.82	1.25
Fear/Dread	0.06	-1.12	1.23
Mundane	-0.18	-1.40	1.04
Warmth-Comfort	-0.19	-1.34	0.96
Familiarity	-0.32	-1.41	0.78
Immersion	-0.41	-2.13	1.32
Visual	-0.46	-1.75	0.83
Identity/Form	-0.49	-1.53	0.54
Outside	-1.15	-3.36	1.07
Multiple - Single	-1.38	-2.55	-0.21
Knowing/Feeling	-1.58	-2.76	-0.41

Table 5 – Odds ratios by diagnosis in study 1

	logOR	LoCI	HiCI
Auditory/Verbal	0.95	-0.12	2.01
Mundane	0.76	-0.40	1.92
Visual	0.45	-0.75	1.64
Interaction	0.33	-1.09	1.75
Purpose	0.27	-0.95	1.49
Stress-Illness	0.25	-0.87	1.36
Inside	0.24	-0.75	1.23
Identity/Form	0.14	-0.85	1.13
Grief/Bereavement	0.09	-1.00	1.18
Outside	0.07	-1.61	1.75
Sleep	-0.04	-1.06	0.98
Multiple - Single	-0.15	-1.15	0.85
Familiarity	-0.46	-1.51	0.58
Multiple- Various	-0.46	-1.51	0.58
Spiritual	-0.51	-1.75	0.74
Tactile	-0.59	-1.75	0.57
Fear/Dread	-0.59	-1.75	0.57
Smell	-0.66	-3.11	1.79
Being Watched	-0.73	-2.21	0.75
Personal Space	-0.73	-1.88	0.41
Warmth-Comfort	-0.87	-2.01	0.27
Knowing/Feeling	-0.92	-1.94	0.09
Immersion	-1.90	-4.08	0.28

Table 6. Qualitative examples for each code analysed

Auditory: "A nagging feeling that I am being followed or watched, occasionally hearing movement or as if there is someone directly behind me or closer to me than anyone else is around me. Feeling as if my granddad was there (many years after his death) and was watching me. It felt like a warm presence and I could smell him."

Visual: "During a bright sunny morning and in a happy mood I was walking down stairs at home and felt someone behind me. I turned to look and saw a monk dressed in brown habit with a cowl over his head. I have many other experiences of the presence of people (sometimes where I worked, sometimes at other people's houses and even whilst in the car."

Tactile: "I awoke from sleep to feel the presence of my late ex-boyfriend standing in the middle of the room. He was not visible to me, but I knew he was there, and that it was him rather than anyone else. About 18 months later, I felt his presence in bed beside me. Again I could not see him, but I could feel the pressure of his body along the length of mine."

Smell: "Feeling as if my granddad was there (many years after his death) and was watching me. It felt like a warm presence and I could smell him."

Personal Space: "The feeling is akin to smoke, albeit concentrated near the skin surface. Sometimes, though, it can feel like strong air currents coming out of the skin and extending beyond the body. At other times, it's like a pillow. Occasionally, I feel like this person-presence is standing next to me, 'looking' over what I'm doing. But then I would quickly look away, as it feels uncomfortable."

Fear/Dread: "A feeling of utter terror, as if this presence had the power to hurt me."

Warmth/Comfort: "It varies greatly depending on the circumstance, but it is very rarely a negative feeling. It is generally a feeling of comfort, of being loved or protected, spiritual awakening or artistic inspiration."

Familiarity: "I know the person whose presence I experience. She is a young woman with her own problems and life story which is completely different from and separate from me and my life, yet we are linked. Sometimes there are words, images and always emotions that I pick up from her."

Identity/Form: "I've felt/ sensed someone's age, physical build & gender. Sometimes I've been aware of feeling physically warmer."

Being Watched: "A feeling of being watched, that someone else is in the room with me when no one else is there."

Purpose: "I had succumbed to hypothermia in a race and had basically decided to sleep in some marshalls and give up. Something told me to open my eyes and ahead I saw a light. I sprung back into life and found my way"

Knowing/Feeling: "There is an awareness of something else being present, that cannot be explained."

Grief/Bereavement: "When my cousin died I was aware of her presence at the funeral during the service, it felt like being bathed in a warm golden light although I couldn't see any. It seemed to come from the front of the chapel. I could sense it was my cousin. When I got home I could feel her there all weekend, above and the left of the chair where I sit, over by the wall. It was good to feel her there - it was amazingly strong feeling, not a slight feeling."

Stress/Illness: "I was staying in my mother's house after she was transferred to a home. She had been suffering from Alzheimer's… I grew up in that house and was very familiar with its energy. I felt

anxious at night, the energy in pretty much every room was pretty awful and it felt like something was lurking but I couldn't pinpoint exactly where and what."

Multiple – Single Context: "If it ever happens I just feel like there is someone in the same room as me, usually this is when it's dark and my vision cannot detect everything in the room so I guess my mind is trying to guess what is there and gives the false perception of someone being there"

Multiple – Varied Contexts: "It happens in many different types of situations. Sometimes at home, at other people's homes, at school, with other people, sometimes other people feel it too"

Immersion: "When meditating, sitting in the quiet, and sometimes just at random times. The last time I was sat at my desk concentrating on my work and I felt spirit touch me at the back of my head. it's a comforting experience, not at all disturbing."

Interaction: "I am aware of a presence very close, standing next to me and then blending their energy with mine (moving in) if that is how they wish to communicate."

Spiritual: "Usually it is during prayer or in worship either in a group situation or on my own. I'll be speaking to God and will just feel tingly all over - especially head /arms / hands and tummy."

Sleep: "A few times before falling asleep I have felt something close by, this has left me in a state of fear when I then couldn't move or fall asleep as I felt the need to protect myself."

Mundane: "It's so common for me I don't really think about it. Sometimes it's annoying to be bothered by these presences."

Inside: "An unknown (maybe angelic) presence in my room before I entered it. It was quite obvious to me at the time that it occurred when my door was open just at the right angle, and through the crack at the hinge."

Outside: "When I go running I sometimes sense something is behind me or about to leap out of the woods, even when there's silence. I sometimes turn around but never see anything. My friend tells me the forest has "a lot of energy" whatever that means, but I assume it's my imagination. Sometimes I feel a huge looming presence behind me or to my sides. We don't have large predators out here but sometimes I imagine a large predator, more often I simply feel an energy following me."

Table 7. FP ratings for studies 2 and 3

**A. Study 2**	**Frequency (%)**				
	Never	*Hardy Ever*	*Rarely*	*Occasionally*	*Frequently*
**a. MUSEQ FoP subscale**					
i) I felt the presence of someone, even though I could not see them (e.g., behind me, or in another room).	4.3	0	8.5	23.4	63.8
ii) I have felt an unseen evil presence around me.	45.7	32.6	6.5	13.0	2.2
iii) I have felt an unseen angelic presence around me.	15.6	22.2	20.0	17.8	24.4
iv) I have felt the presence of a relative or friend who has passed away.	0	15.2	10.9	19.6	54.4
**b. Qualities of presence (PatientsLikeMe)**					
v) Do you feel as though this presence is human?	*Human*	72.3	*Non-human*	27.7	
vi) Is the presence familiar to you?	*Yes*	84.8	*No*	15.2	
vii) Does the presence ever speak to you or make another noise?	*Yes*	71.7	*No*	28.3	
viii) Does the presence ever touch you?	*Yes*	52.2	*No*	47.8	
ix) Are these experiences always the same?	*Yes*	17.0	*No*	83.0	

B. Study 3	Frequency (%)				
	Never	*Hardy Ever*	*Rarely*	*Occasionally*	*Frequently*
**a. MUSEQ FoP subscale**					
i) I felt the presence of someone, even though I could not see them (e.g., behind me, or in another room).	28.6	27.4	11.9	25.0	7.1
ii) I have felt an unseen evil presence around me.	64.3	1.2	25.0	6.0	1.2
iii) I have felt an unseen angelic presence around me.	59.5	16.7	9.5	11.9	1.2
iv) I have felt the presence of a relative or friend who has passed away.	56.0	21.4	9.5	9.5	2.3
**b. Qualities of presence (PatientsLikeMe)**					
v) Do you feel as though this presence is human?	*Human*	79.4	*Non-human*	17.5	
vi) Is the presence familiar to you?	*Yes*	54.8	*No*	45.2	
vii) Does the presence ever speak to you or make another noise?	*Yes*	19.1	*No*	80.1	
viii) Does the presence ever touch you?	*Yes*	8.1	*No*	92.9	
ix) Are these experiences always the same?	*Yes*	45.0	*No*	55.0	

Table 8. Odds ratios between experiments 1 & 2 (A), 1 & 3 (B), 2 & 3 (C)

A.	logOR	LoCI	HiCI
Spiritual	1.79	0.93	2.65
Immersion	1.47	0.47	2.48
Multiple- Various	1.27	0.46	2.08
Inside	1.02	0.21	1.84
Interaction	0.86	-0.11	1.83
Personal Space	0.78	-0.04	1.60
Knowing/Feeling	0.77	-0.02	1.57
Purpose	0.64	-0.24	1.52
Tactile	0.58	-0.26	1.41
Warmth-Comfort	0.45	-0.37	1.28
Outside	0.45	-0.75	1.65
Visual	0.32	-0.57	1.22
Identity/Form	0.27	-0.52	1.06
Familiarity	-0.08	-0.90	0.74
Auditory/Verbal	-0.42	-1.28	0.44
Grief/Bereavement	-0.54	-1.48	0.40
Multiple - Single	-0.69	-1.54	0.16
Smell	-0.73	-3.02	1.57
Sleep	-0.90	-1.82	0.02
Fear/Dread	-1.18	-2.35	0.00
Stress-Illness	-1.57	-2.87	-0.28
Being Watched	-1.93	-4.03	0.18

B	logOR	LoCI	HiCI
Outside	2.93	1.90	3.96
Purpose	0.61	-0.26	1.47
Being Watched	0.37	-0.64	1.38
Multiple - Single	0.34	-0.42	1.10
Visual	0.20	-0.68	1.09
Warmth-Comfort	-0.02	-0.86	0.82
Smell	-0.10	-1.93	1.73
Grief/Bereavement	-0.13	-0.98	0.72
Familiarity	-0.21	-1.02	0.60
Personal Space	-0.29	-1.18	0.60
Knowing/Feeling	-0.32	-1.09	0.46
Fear/Dread	-0.34	-1.26	0.58
Identity/Form	-0.38	-1.14	0.38
Sleep	-0.38	-1.20	0.43
Tactile	-0.64	-1.63	0.34
Interaction	-0.88	-2.24	0.49
Stress-Illness	-0.90	-1.93	0.12

Multiple- Various	-0.94	-1.86	-0.02
Inside	-1.12	-1.97	-0.27
Auditory/Verbal	-1.28	-2.28	-0.28
Immersion	-1.73	-3.86	0.40
Spiritual	-1.75	-3.29	-0.20

**C**	logOR	LoCI	HiCI
Outside	2.48	1.43	3.53
Being Watched	2.30	0.19	4.41
Multiple - Single	1.03	0.13	1.92
Fear/Dread	0.84	-0.42	2.10
Stress-Illness	0.67	-0.78	2.12
Smell	0.62	-1.81	3.06
Sleep	0.51	-0.48	1.51
Grief/Bereavement	0.41	-0.60	1.42
Purpose	-0.03	-0.91	0.86
Visual	-0.12	-1.06	0.81
Familiarity	-0.13	-1.02	0.76
Warmth-Comfort	-0.48	-1.37	0.42
Identity/Form	-0.65	-1.49	0.19
Auditory/Verbal	-0.85	-1.95	0.24
Personal Space	-1.07	-2.00	-0.15
Knowing/Feeling	-1.09	-1.95	-0.23
Tactile	-1.22	-2.23	-0.21
Interaction	-1.74	-3.08	-0.39
Inside	-2.14	-3.10	-1.19
Multiple- Various	-2.21	-3.19	-1.22
Immersion	-3.20	-5.28	-1.13
Spiritual	-3.54	-5.08	-2.00

Figure 1 DES Derealisation Scores in studies 1 (A), 2, (B), and 3 (C).

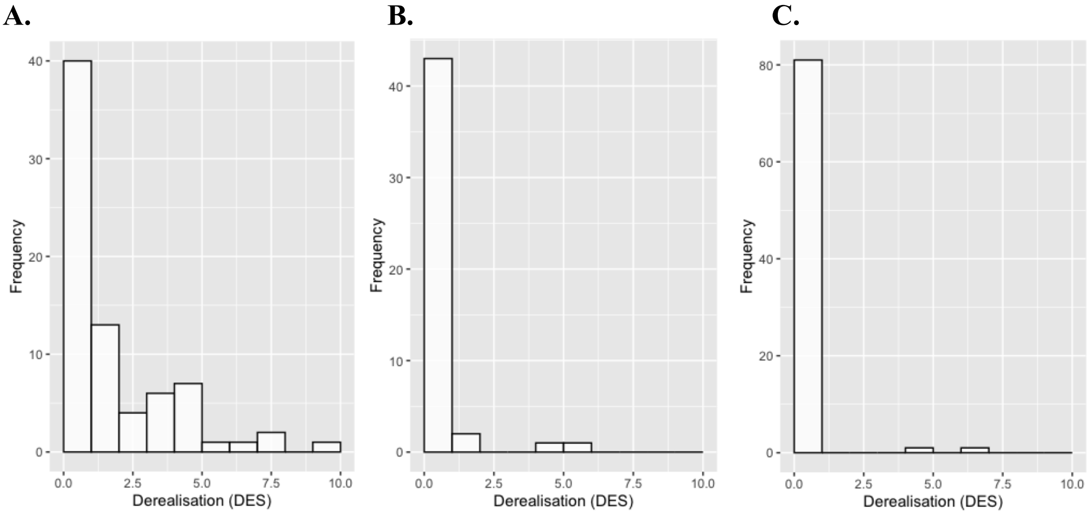

<u>Table 9: Correlation matrix for study 2</u>

	LSHS	Paranoia Checklist	VISQ Dialogic	VISQ Other	SCI
Felt Presence (MUSEQ)	0.44**	0.23	-0.04	0.29	-0.10
LSHS	1	0.23	0.25	0.53***	-0.22
Paranoia Checklist	-	1	0.06	0.27	-0.35*
VISQ Dialogic	-	-	1	0.57***	-0.31*
VISQ Other	-	-	-	1	-0.32*

<u>Table 10: Correlation matrix for study 3</u>

	LSHS	Paranoia Checklist	VISQ Dialogic	VISQ Other	SCI
Felt Presence (MUSEQ)	0.44***	0.11	0.11	0.27*	-0.29**
LSHS	1	0.36***	0.18	0.45***	-0.28**
Paranoia Checklist	-	1	0.24*	0.36***	-0.24*
VISQ Dialogic	-	-	1	0.50***	-0.29**
VISQ Other	-	-	-	1	-0.24*

Table 11. Regression results for study 2

		Beta	SE	*B*	t	p	Low CI	High CI	F	df	adj. R2	p
**Model 1**	Diagnosis	2.32	1.30	0.28	1.79	0.080	-0.02	0.58	3.64	3,41	0.15	0.020
	LSHS	0.33	0.15	0.31	2.27	0.029	-0.05	0.68			**AIC:**	223.04
	Paranoia	-0.01	0.09	0.04	-0.16	0.876	-0.33	0.41				
**Model 2**	Diagnosis	2.40	1.32	0.29	1.82	0.077	-0.01	0.59	2.31	6,38	0.15	0.054
	LSHS	0.30	0.19	0.30	1.57	0.124	-0.16	0.77			**AIC:**	225.69
	Paranoia	-0.02	0.10	0.07	-0.16	0.872	-0.32	0.47				
	VISQ-Dialogic	-0.15	0.09	-0.36	-1.68	0.102	-0.74	0.02				
	VISQ-Other	0.11	0.12	0.23	0.99	0.329	-0.29	0.74				
	SCI	0.02	0.09	0.09	0.21	0.835	-0.26	0.45				

Table 12. Regression results for study 3

		Beta	SE	*B*	t	p	Low CI	High CI	F	df	adj. R2	p
**Model 1**	Education (High School)	1.94	1.41	0.20	1.38	0.173	-0.09	0.49	7.78	5,78	0.29	<0.001
	Education (Postgraduate)	0.33	1.28	0.04	0.26	0.796	-0.29	0.38			**AIC:**	427.79
	Education (Undergraduate)	0.33	1.23	0.05	0.26	0.792	-0.30	0.40				
	LSHS	0.56	0.11	0.54	5.10	0.000	0.33	0.75				
	Paranoia	-0.03	0.04	-0.06	-0.60	0.547	-0.27	0.15				
**Model 2**	Education (High School)	2.25	1.44	0.23	1.57	0.122	-0.06	0.53	5.11	8,75	0.28	<0.001
	Education (Postgraduate)	0.65	1.31	0.09	0.50	0.620	-0.26	0.43			**AIC:**	431.22
	Education (Undergraduate)	0.66	1.26	0.09	0.52	0.604	-0.27	0.45				
	LSHS	0.49	0.13	0.48	3.71	0.000	0.22	0.73				
	Paranoia	-0.04	0.05	-0.10	-0.88	0.384	-0.32	0.12				
	VISQ-Dialogic	-0.04	0.06	-0.07	-0.67	0.505	-0.29	0.14				
	VISQ-Other	0.06	0.11	0.08	0.61	0.547	-0.19	0.35				
	SCI	-0.07	0.05	-0.14	-1.41	0.164	-0.35	0.06				
